# Adoptable Interventions, Human Health, and Food Safety Considerations for Reducing Sodium Content of Processed Food Products

**DOI:** 10.3390/foods7020016

**Published:** 2018-02-01

**Authors:** Abimbola Allison, Aliyar Fouladkhah

**Affiliations:** Public Health Microbiology Laboratory, College of Agriculture, Human and Natural Sciences, Tennessee State University, Nashville, TN 37209, USA; abimbolaallison20@gmail.com

**Keywords:** adoptable intervention, excess dietary sodium, sodium reduction, food safety, processed foods

## Abstract

Although vital for maintaining health when consumed in moderation, various epidemiological studies in recent years have shown a strong association between excess dietary sodium with an array of health complications. These associations are robust and clinically significant for development of hypertension and prehypertension, two of the leading causes of preventable mortality worldwide, in adults with a high-sodium diet. Data from developed nations and transition economies show worldwide sodium intake of higher than recommended amounts in various nations. While natural foods typically contain a moderate amount of sodium, manufactured food products are the main contributor to dietary sodium intake, up to 75% of sodium in diet of American adults, as an example. Lower cost in formulation, positive effects on organoleptic properties of food products, effects on food quality during shelf-life, and microbiological food safety, make sodium chloride a notable candidate and an indispensable part of formulation of various products. Although low-sodium formulation of each product possesses a unique set of challenges, review of literature shows an abundance of successful experiences for products of many categories. The current study discusses adoptable interventions for product development and reformulation of products to achieve a modest amount of final sodium content while maintaining taste, quality, shelf-stability, and microbiological food safety.

## 1. Human Health, Excess Dietary Sodium, and Consumption Recommendations

Sodium is an essential nutrient for maintaining health. Consumption of higher than recommended values, however, have been associated with various health complications. Excess sodium intake is one of the main contributing factors to the development of hypertension in adults. It is estimated that around 26% of people around the globe are currently suffering from hypertension [1]. In the United States, life-time probability of developing hypertension is about 90% [1]. It is estimated that as many as 16.7 million individuals die every year around the world, including around 850,000 people in the United States, as the result of cardiovascular diseases. Around eight million of these premature deaths are associated with hypertension in adults, and many additional are directly related to pre-hypertension. In the United States, as many as 27% of adults suffer from hypertension with an additional 31% in the prehypertension stage [2,3]. It is also estimated that less than 1% of American adults meet the joint dietary guidelines for sodium and potassium [4]. It is noteworthy that some studies indicate the association of sodium intake with morbidity and mortality is a “J shape” in nature, meaning that direct and strong associations exist at high levels (over 4 g of salt per day) and at very low levels (less than 2 g of salt per day). Another notable misconception is that “sodium content and salt content are nutritionally the same”. In fact, sodium, is part of sodium chloride (salt) and based on the molar mass, sodium content multiplied by 2.5 is equal to sodium chloride content. In other words, sodium content of a diet is 40% of the total salt intake [5]. Genetics also play a critical role in an individual’s susceptibility to elevated sodium intake in diet and hypertension. Nevertheless, pre-clinical, clinical, and epidemiological investigations in recent decades have shown robust and clinically significant associations among an array of health complications for salt consumptions of more than 4 g (>1600 mg sodium) per day. Blood vessels, the heart, kidneys, and the brain could be adversely affected with high salt consumption [6]. Excess dietary sodium had also been associated with an increased likelihood of developing gastric cancer in case-control and cohort studies [7], increased likelihood for development of overeating habits, obesity, renal diseases, and asthma [8], and significantly increased risk of stroke [9]. Comprehensive review of various ecological, cross-sectional, case-control, and prospective studies by the European Food Safety Authority had also shown negative effects of excess dietary sodium on bone health and various components of cardiovascular function [10].

The recently updated guideline of the World Health Organization (WHO) recommends adults consume less than 2000 mg of sodium (<5 g of salt) per day. It further recommends consumption of at least 3510 mg of potassium every day [11]. The United States Department of Agriculture (USDA) dietary guidelines similarly recommend 2300 mg sodium per day as the “Tolerable Upper Intake” Level (the highest amount that is likely to pose no immediate health concern) for persons aged ≥14 and less for those 2–13 years. It further recommends “Adequate Intake” of 1500 mg per day for 5 to 50-year-old individuals [12]. In the United States, around 90% of adults consume sodium in levels higher than recommended amounts [11,12], on average 3400 mg sodium per day. Sodium intake in European populations is estimated to be 3000 to 5000 mg per day as well [13]. 

## 2. Main Contributors to Dietary Sodium and Sodium Reduction Initiatives

The vast majority of natural foods contain a moderate amount of sodium. As an example, milk and egg contain 50 and 80 mg/100 g sodium, while processed foods such as bread, bacon, pretzel, soy sauce, and stock cubes could have sodium contents as high as 250, 1500, 1500, 7000, and 20,000 mg/100 g, respectively [11]. Dietary guidelines of USDA estimate as high as 75% of dietary sodium could be associated with consumption of processed and pre-packaged food products in the United States [12]. Other studies also estimate around 77% of sodium is associated to consumption of processed foods [3]. In the European Union, similarly, 70–75% of intake is associated with processed foods, 10–15% to natural foods, and 10–15% to discretionary salt added to meals [13]. Breads, processed meat, cheeses, and spreads are the dominant contributors of sodium in Western diets, accounting for up to 37% of an adult’s sodium intake [14]. High sodium processed foods appear to be a concern for individuals of all ages. As an example, about 90% of food advertisements on Saturday morning children’s TV programs are foods high in sodium content [15]. The most recent epidemiological study by the Centers for Disease Control and Prevention (CDC), which analyzed 14728 participants of ≥2 years of age, indicates 89% of American adults and over 90% of children exceed the recommended amount of sodium [16]. Analyses of 44,000 food purchases by 21,000 households, concludes that reducing the sodium content of a small number of high-sodium food categories could yield the largest decreases in sodium available to the public [14]. 

Studies investigating the sodium intake at the population level show an average worldwide sodium intake of 2300 to 4600 mg per day. Considering the health complications associated with high sodium diet and the prevalence of hypertension and prehypertension in the United States and around the globe, reducing the dietary sodium intake is a public health necessity and an ethical duty of food manufacturers in private industry. Recent analyses of world-wide endeavors indicate that a gradual reduction of salt in processed foods would go unnoticed in most cases by the vast majority of consumers, and it could ultimately reduce each individual’s intake of sodium [17]. The challenge for assessing efficacy of national programs is creating precise and accurate methodologies for determining the intake of sodium consumed by individuals at the population level to avoid under-reporting and under-estimation. Epidemiological tools such as twenty-four-hour food frequency and urinary collection as well as methods such as spot urine sample analyses [18] and “one-week salt estimation method” [19] are common and emerging practices to monitor and estimate the intake of sodium at the population level. 

As a means to reduce the non-communicable diseases burden associated to high sodium diets, many nations have mandated successful interventions in last few decades. The Japanese experience (1960–1970) and the current program existing since 1975 in Finland are two notable examples of classic population-wide interventions [8]. With approximately 13.5% of worldwide deaths associated with hypertension and high sodium diet, many recent endeavors have also been implemented around the globe [20,21,22]. The World Health Organization has also recently harmonized a global recommendation, proposing to reduce at least 30% of sodium intake by 2025 in various populations around the globe [23]. In the United States, the Institute of Medicine also proposed similar reduction strategies with special emphasis on interventions conducted in close harmony with food processing and restaurant establishments [24]. 

Studies indicate the American population-wide experience has not been efficacious in recent years, with only 0.015% of American adults currently meeting the joint dietary guidelines for sodium and potassium intake [4]. Canadian health authorities have initiated similar evolving programs since 2006 [25]. One major concern of the program is inaccuracies on the food product’s sodium labels in approximately 18.4% of tested samples in a four-year prospective study [26,27]. A 10-year retrospective study following the sodium content of nine key food groups in New Zealand also reveals no overall statistically significant reductions in the products from 2003 to 2013 [28,29]. A recent cross-sectional study in China also revealed a vast majority of families consume more than the recommended amount of sodium. Contrary to Western diets, it is estimated that up to 90% of sodium intake in the Chinese diet is from meals and foods prepared at home, and around 10% from processed and/or pre-packaged foods [30]. The United Kingdom (UK) Food Standards Agency have implemented a nationwide program for reducing the sodium intake in the nation, a successful program that has been adopted in other nations and involves consumer awareness campaigns and close collaboration with the private food industry [31]. Similar programs in the European Union are targeting <5 g/day of dietary salt intake (equivalent to <2 g/day of sodium intake). About half of the European member states have made recent legislative changes for taxation, mandatory nutrition labeling, and/or regulated health/nutrition claims [32,33]. South Africa has also recently introduced nationwide legislation to limit the sodium of an array of processed products. Many food products in the country are currently meeting permitted upper levels and around 25% are still above the maximum limit of the legislation [34]. Further information about global initiatives have been recently discussed by Trieu et al. [35].

## 3. Adoptable Interventions for Reducing the Sodium in Processed Foods

Studying various commodities in Western diets, recent studies emphasize that breads, processed meats, cheeses, sauces, and spreads have salt content considerably higher than reasonable benchmarks, suggesting reformulation for such high sodium categories [36]. While there are various proposed interventions for reducing the sodium content of the processed food, many of the solutions require extensive initial capital investments or propose exploratory use of compounds that have not received regulatory status as a food additive. The current study provides a summary of the adoptable interventions and food safety considerations, with an emphasis on high risk food categories and those interventions with immediate and adoptable applications.

### 3.1. Reducing the Particle Size 

As a topical seasoning agent for low moisture foods, such as potato fries and chips, some studies suggest use of salt crystals with reduced particle sizes (5–20 microns). They observed up to 30% reductions in sodium content of solid foods due to higher solubility of salt particles in saliva, and thus higher intensity of flavor [37,38]. Similar studies described low-particle-sized salt and salt alternatives for application on popcorn, breaded frozen foods, crackers, cheeses, potato fries, spreads, and cereal bars [39,40,41]. Other studies, suggest using a mixture of salt and alternative salts with small, medium, and large particle sizes (i.e., 15, 75, and 220 microns, respectively) and reported a 33% reduction in sodium content of potato chips using such mixtures [42,43]. 

### 3.2. Application of Salt Alternatives and Bitter Blocking Agents

A sodium-free aqueous solution with acceptable saltiness perception was developed using potassium, calcium, and magnesium salts with natural bitter blocking agents such as organic acids [44,45]. A sensation similar to sodium chloride was similarly produced by using a mixture of potassium citrate, calcium citrate, and citric acid in deionized water [46]. Low-sodium vegetable juices [47] and chicken soups [48] were formulated successfully using potassium salts, yeast extract, hydrolyzed fish and vegetable proteins, and organic acids as bitter blocking ingredients. Sugar alcohols, monosodium glutamate, potassium chloride, sucrose, sodium inosinate, and guanylate are other ingredients successfully utilized in various low-sodium formulations [49]. Up to 60 and 100% reductions of sodium were also achievable in chicken broth using different ratios of potassium chloride, magnesium chloride, and magnesium sulfate. A mixture of potassium chloride, ammonium chloride, sucrose, sodium inosinate, guanylate, and organic acids has been also used successfully for low-sodium reformulations [50]. Monovalent and divalent chloride salts, particularly potassium chloride, are the most extensively investigated sodium chloride alternatives. Many other salts such as lithium-, choline-, and ammonium-based compounds have also been utilized for low-sodium reformulations with uses that are exploratory in nature and pending regulatory approval as a food additive [51].

### 3.3. Natural Flavor Enhancers 

A sodium-free salt substitute formulation was developed using natural flavor enhancers such as onion powder, ginger oil, and rice flour [52,53]. Similar low-salt formulations were prepared using natural flavor enhancers such as skim and whole milk powder, buttermilk powder, yeast extract, and rice flour [54]. Leak, onion, and chive powders were also suggested as natural flavor enhancers in formulation of low-sodium products [55]. Tomato solids, whey proteins minerals, and powdered vinegar have also been successfully utilized for reformulation of products with up to 50% less sodium [56,57,58]. Hydrolyzed proteins of soy, fish, shellfish, and various plant proteins have, as well, been utilized in dry salt formulations with up to 45% less sodium [59]. A naturally driven seaweed derivative has also been shown to have salty perception with 90–99% less sodium content [60,61,62]. Use of about 5 to 10% distillers’ grain with solubles, a byproduct of biofuel production, has also shown to be effective in reducing the sodium content of cheddar cheese sauce, pizza crusts, breads, and vegetarian burger mixes [63]. Ingredients naturally containing glutamic acid, such as various hydrolyzed proteins, rice flour, and soy-based ingredients, and those containing other salts of amino acids and nucleotides, such as yeast extracts and tomato powder, are among the most investigated natural flavor enhancers for reducing the sodium content of various formulations [51].

### 3.4. Heterogeneous Distribution of Salt and Use of Aroma Compounds 

Heterogeneous distribution of salt in a formulation has been proposed for creating a “sensory contrast” and for providing saltiness perception with reduced amount of sodium in a formulation. One study reported a 28% decrease in salt without noticeable sensory changes in baked products by spatial heterogeneity of salt in formulation [64]. They observed minimal migration of salt in such baked products and observed the highest saltiness enhancement with increased magnitude of sensory contrast. Similar conclusions were obtained in studies of cream-based multi-layer savory pastries. Heterogeneous distribution of salt resulted in products with comparable sensory perceptions while containing 25% less sodium in formulation [65]. A similar study also showed up to a 42% reduction in sodium content of cereal- and cream-based multi-layer products by heterogeneous distribution of salt in the products [66]. 

Use of aroma compound has also been explored for enhancing the saltiness of a product without use of alternative salts and flavor enhancers. The aroma-taste interaction, particularly the effects on saltiness perception, have been studied using sensory panels coupled with brain imaging studies [51]. Use of compounds isolated from beef broth, as an example, have been reported to enhance the saltiness perception of liquid foods, and the aroma associated with ham has been proposed to increase the salt perception of savory cereal-based snacks [65]. The odor of soy sauce has also been associated with increased saltiness perception, with enhanced results in approximately 40% of the panelists compared to the remaining panelists of taste panel [67].

## 4. Product Specific Experiences

As one of the most economically priced ingredient in food product development, sodium chloride is an integral part of food manufacturing for its properties in microbiological safety and quality during shelf-life of an array of products. Many food categories such as dry-cured meat products rely on sodium chloride as one of the microbiological preservation methods coupled with other hurdles such as low water activity, low pH, and/or refrigeration. Many other products such as breads, emulsions, sausages, and spreads rely on the technological properties of sodium chloride for improved taste, texture, color, and consumers’ acceptability [1]. Inevitably, reformulation of products for reducing sodium content will pose unique sensory, microbiological, and quality challenges.

### 4.1. Processed Meats

Processed meats are a dominant source of salt in diets. Sodium chloride plays a crucial role for final taste, texture, color, microbial safety, shelf stability, consumer acceptability, and economic formulations of various processed meat products [1,68]. Studies have shown potassium, calcium, and magnesium chlorides could be an affordable and efficacious alternative to sodium chloride in different meat products. Although comparable formulations could be developed using the above-mentioned salt mixtures, certain changes are unavoidable during the processes to optimize the organoleptic properties of the final product. As an example, using a combination of sodium and potassium chlorides would require about a 32% increase in post-salting ageing in dry-cured ham. Similar formulations with a combination of sodium, calcium, and magnesium salts would require 52% longer aging relative to the original formulation of dry-cured ham [69]. Similar studies have also explored the use of potassium chloride and sodium triphosphate to achieve a 58 to 77% reduction in sodium content of lean sausages as compared to commercially available products [70]. A study also explored replacement of sodium chloride by potassium chloride in dry-cured bacon. They observed undesirable sensory and volatile characteristics associated with replacements higher than 40% while showed comparable characteristics with the original formulations when the salt was replaced at lower concentrations [71]. In another study, dry-cured ham was also reformulated successfully with 50% less sodium, using potassium chloride alone and in combination with calcium and magnesium chlorides [72]. Similar achievements were reported for development of ready-to-eat desalted cod [73] and dry-cured loins [74]. 

Emerging technologies have been proposed to reduce the sodium content of processed meat. As an example, application of high pressure processing has been shown to yield surimi gel with desirable properties while having only 10% of the sodium content of the original formulation [75]. Application of salt alternatives alone have also been shown to be effective in reducing sodium content of surimi [76]. Similar approaches, by using pulse pressure-assisted brining, have been proposed for processing of bacon [77] and low-sodium turkey breast deli cuts [78]. These studies are still in the developing stages and do not discuss commercial feasibility and the cost associated with utilization of the elevated hydrostatic pressure. Effects of prebiotic ingredients such inulin, poly dextrose, and resistant starch have also been proposed for reformulation of low-fat meat emulsions without appreciable negative effects on batter and final sausage properties [79]. Other studies showed use of phosphate could improve the texture and taste of ground beef patties and eliminate up to 60% of the sodium content of the product after cooking. A study investigating the shelf-life of reformulated products also examined the microbial safety of low-sodium meat-containing ready-to-eat meals. It concluded that up to a 50 to 66% salt reduction may still yield a product with similar microbiological safety during shelf-life and after thawing at 4 and 25 °C [80].

Similar studies also concluded that potassium chloride at a one-to-one ratio with sodium chloride has the same antimicrobial properties as sodium chloride alone and thus could be used interchangeably in formulation of low-sodium meat products [81]. 

Recent review studies indicate that although salt reduction in many meat products is achievable, it requires product specific research and development for assuring the safety and organoleptic properties expected by consumers and regulatory agencies [82]. Others concluded that salt content of around 2% in general, 1.4% in cooked sausages, and 1.75% in lean meat products appears to be a threshold of acceptability by consumers [83]. Current adoptable interventions are particularly revolving around the use of potassium chloride as a partial replacement for sodium chloride in addition to using flavor enhancers, bitter blocking agents, and non-potassium salt alternatives [84].

### 4.2. Dairy Products and Cheeses

As one of the main contributors to excess dietary sodium, reducing the sodium content of cheese products has been investigated extensively in recent years [1]. In a study exploring replacement of sodium chloride with calcium, magnesium, and potassium chlorides, products with reduced sodium of up to 0.7% were rated favorably by panelists with some notes on sour residual taste and after taste in formulations with the lowest sodium chloride content [85]. Similar studies were conducted for re-formulation of cheese products with 0.5 to 1.5% salt. They observed comparable panelists’ texture perception, instrumental texture properties, and similar aroma for low-sodium products and the original formulations, particularly for products containing full fat content. They reported less favorable sensory perceptions when both sodium and fat were reduced simultaneously in a re-formulation [86]. A major challenge for production of reduced sodium cheese appears to be acidic flavor/aftertaste development due to excessive acid production by the starter culture in absence of sufficient sodium during production. It has been reported that application of novel intervention such as elevated hydrostatic pressure could reduce the initial starter culture bacterial load, thus improving the final organoleptic properties [87]. Application of bitter blocking ingredients such as chymosin has also been reported to improve sensory properties of the final cheese products when sodium chloride is replaced with alternative salts [88]. Others have also reported the addition of arginine to improve the quality of reduced-sodium cheese and probiotic dairy products. They achieve up to 50% replacement of salt with potassium chloride, with up to 1% of added arginine [89]. In a study including 270-day ripening of cheddar-style cheese, reducing sodium from 1.9% to 1.2% and 0.9% resulted in increased moisture, lactic acid, and reduced rate of starter culture die-off during ripening. The undesirable sensory properties were enhanced in reformulation of products with simultaneous reduction of sodium and fat content, particularly with products having 22% or 16% fat, compared to 33% [90]. 

A recent study concluded that a 15% reduction in sodium content of cheeses could be achieved without concerns of consumers’ rejection. Presence of nutritional claims such as sodium-free (less than 5 mg per labeled serving), low-sodium (≤140 mg per labeled serving), or reduced in sodium (at least 25% less sodium than appropriate reference food) have also been associated with increased willingness of consumers to purchase cheese products [91]. Recent studies show success of experiments for re-formulation of low-sodium products by moderate replacement of sodium chloride with potassium chloride. Such an approach could lead to 25%, 13%, and 67% reductions in sodium content of cheddar, mozzarella, and processed cheese, respectively, with minimal sensory sacrifices, while higher reductions could lead to a metallic after taste and off-notes, typically associated with potassium chloride [92]. Use of prebiotics and phosphate and optimizing intrinsic factors of the formulation, particularly fat content, are remaining critical steps for achieving success in formulation of a low-sodium processed products [93].

### 4.3. Breads and Baked Goods

Contrary to many consumers’ beliefs, bread and baked products are one of the main contributors to excess dietary sodium [1,2]. A review of the literature shows an abundance of successful experiences for reduced- and low-sodium reformulation of these products. Studies have been reported that up to a 30% reduction of sodium content and replacement of up to 10% wheat flour with soy flour (that is naturally high in potassium) could lead to final products with comparable sensory properties relative to standard formulations [94]. Similar studies show a reduction of 10% and 20% of salt in bread and flour-based formulations. These reductions did not change organoleptic properties and intent to purchase rated by a sixty-five-member taste panel [95]. Others reported minimal sensory changes, as rated by adolescent and adult consumers, after reformulation of bread with 50% less sodium as well as 50% increased fiber [96]. Similar conclusions were reported [97], when sodium of a flour mix was reduced by 0, 25, 50, and 100% by using a mixture of calcium, potassium, and magnesium salts. These studies, articulated the possibility of reformulation of low-sodium products with minimal effects on texture, taste, quality attributes, and shelf-life [1]. Further consumer testing found that up to a 30% reduction in sodium content of breads is achievable without affecting the consumers’ acceptability of the products [98]. As further discussed in Section 3.1 and Section 3.4, topical application of salt, use of salt with reduced particle sizes for such applications, and heterogeneous distribution of salt in baked products could also meaningfully reduce the sodium content of the products. 

### 4.4. Soups, Dressings, and Ready-to-Eat Meals

Use of monosodium glutamate and calcium diglutamate has been shown to be an effective alternative for reducing the sodium content of soups. Both compounds have shown to improve saltiness perception in the presence of a small amount of sodium in the food matrix and have shown effectiveness as a sole replacer of salt or in combination with each other [99]. Up to a 33% sodium reduction was similarly observed by use of calcium diglutamate in chicken broth [100]. Potassium-based salt alternatives, elaborated in Section 3.2, has also shown to be effective in the replacement of the vast majority of sodium content of a soup formulation [46,47,48]. Other studies investigating the preferences of 646 consumers indicated most consumers of soups, particularly for products to be consumed in home environment, would accept products of up to 32% less sodium even when products were formulated without a sodium replacement [101]. In similar settings, it was concluded that up to a 48% reduction of sodium in vegetable soups could be achieved without a change in consumers’ liking or purchase intent [102]. Studies of ready-to-eat meals also showed similar trends, with overall in excess of a 50% salt reduction achievable without depreciation from panelists. Gradual reduction of 40% of salt without any substitution and replacement of up to 60% of salt with potassium chloride and yeast extract were also reported as a successful experience for formulation of ready-to-eat products [103]. In a similar study of frozen ready-to-eat meals, up to 0.3% of salt was replaced successfully with potassium chloride, achieving a product with comparable sensory attributes as the full-salt control [104]. Use of ingredients naturally high in glutamic acid such as rice flour and soy sauce could also enhance the saltiness of complex foods. Salad dressing and tomato soup, as examples, were formulated with 33 to 50% less sodium by replacing portions of the salt with soy sauce powder [105]. Up to 25% reductions were similarly reported in chicken broth, tomato sauce, and coconut curry sauce using soy and fish-based sauces to replace a portion of sodium chloride [106].

## 5. Food Safety and Quality Considerations

Pathogens of public health concern such as various pathogenic serogroups of *Escherichia coli*, serotypes of *Listeria monocytogenes*, serovars of *Salmonella*, species of *Campylobacter*, and strains of *Staphylococcus aureus* and *Clostridium botulinum* could cause food safety challenges in processed food products without the presence of salt in formulation. Spoilage organisms such as various species of *Pseudomonas*, yeasts, and molds could also challenge the success of a low-sodium reformulation [107]. As an example, 3% sodium chloride in a formulation of cooked meat products could inhibit the multiplication of *C. perfringens* [108], and a higher than 3% concentration of NaCl has been shown to inhibit the production of botulism toxin by *C. botulinum* type E [109]. Some pathogenic organisms have enhanced tolerance to salt concentrations, as an example, *Escherichia coli* O157:H7 could multiply in an environment with as high as 6.5% salt, and it could be inhibited by salt concentrations of above 8.5% [110]. Salt tolerant and halophilic bacteria could tolerate even higher concentration of salt; organisms such as *Yersinia enterocolitica*, *Staphylococcus aureus*, and *Listeria monocytogenes* could multiply in the presence of 9% NaCl and survive a 20% concentration of salt for several weeks [109,111].

Although sodium chloride is an integral part of the manufacturing of an array of products, various studies have indicated the antimicrobial properties of sodium chloride are primarily due to its dehydrating capacity [1,112,113]. It is also suggested that sodium chloride’s antimicrobial properties could be additionally due to chlorine ion toxicity to microorganisms [109]. Many salt alternatives, including potassium chloride, have a similar chemical composition, and recent studies show that salt alternatives such as potassium chloride have antimicrobial properties comparable to sodium chloride. A study comparing the microbial profile of original formulations with low-sodium variations concluded that 50 to 66% replacement of sodium chloride with potassium chloride in ready-to-eat meals does not significantly change the microbial load (mesophilic, thermophiles, Coliforms, and *Pseudomonas*) of the product during the shelf-life [114]. A similar study showed comparable antimicrobial properties of sodium and potassium chlorides against *Cronobacter sakazakii*, *Yersinia enterocolitica*, *Staphylococcus aureus*, and *Salmonella* serovars [81]. At the pH ranges of 4.0 to 7.3, it has also been stablished that *Listeria monocytogenes*, a salt tolerant pathogen of concern in ready-to-eat products, exhibits sensitivity to potassium chloride in levels that are nearly identical to sodium chloride [115].

In addition to microbial pathogens, control of spoilage organisms is a crucial aspect of formulation of a product for enhancing shelf-stability and minimizing food waste and economical losses. Similar to pathogenic bacteria, studies exhibited interchangeable antimicrobial properties of sodium and potassium chlorides against spoilage organisms. Two spoilage fungi of concern in bread manufacturing (*Penicillium roqueforti* and *Aspergillus niger*) as an example, exhibited similar multiplication patterns during shelf-life in challenge studies of original full-salt bread formula and the product with a 30% substitution of sodium chloride with potassium chloride [116]. Similar results were also reported during low-sodium reformulation of cheddar cheese where 30 and 50% substitution of sodium chloride with potassium chloride did not affect the multiplication of total molds and yeasts counts in the final product [117]. Although limited information is available in the literature about sensitivity of xerophilic molds, osmophilic yeasts, and halophilic bacteria to potassium chloride relative to sodium chloride, non-halophilic spoilage organisms have comparable sensitivity to sodium and potassium chlorides in an array of products with various intrinsic and extrinsic conditions [1,107,113,118].

Other studies showed products with optimized levels of salt alternatives could have similar quality attributes to the original, full-salt formulations. As an example, low-sodium surimi formulation showed similar thermal denaturation, protein gelation, rheological properties, shear stress, and mechanical fracture values when formulated with salt substitutes [76]. Formulation of aged low-sodium cheddar-style cheese was also reported without any undesirable texture and appearance change after five weeks of aerobic storage [119]. Similar results were also reported during a 25% substitution of sodium chloride with potassium chloride in unaged cheese where no sensory and textural differences were observed due to the substitution [120]. Additional processes could also assure improved quality and safety of new products. As further delineated in Section 4.1 and Section 4.2, extending the aging for dry-cured products and application of elevated hydrostatic pressure for reducing the initial starter load are successful proposed interventions to improve shelf stability and organoleptic attributes of reduced-sodium meat and cheese products, respectively. 

Although nearly all salt alternatives have some reported undesirable sensory characteristics, optimized utilization of salt substitutes could result in no detectable organoleptic changes in the final products. Desalted cod filet, as an example, had been manufactured successfully with 75% lower sodium content, without any undesirable sensory characteristics [73]. After optimization, restructured ham and turkey sausages have also been manufactured successfully with elimination of sodium chloride, with negligible undesirable organoleptic properties [82]. A hash brown mix with 50% less sodium, was also prepared and tested favorably by sensory panelists [121]. Previous review of the literature had shown similar trends [1], exhibiting examples of successful reformulations with minimal changes in sensory properties when salt replacers are utilized at optimized levels [122].

## 6. Conclusions

An abundance of successful experiences in the literature confirm that achieving an appreciably lower sodium content in a wide array of food products is a feasible industrial reality. Main adoptable interventions for sodium reduction are currently revolving around the use of potassium chloride in combination with sodium chloride with or without other non-potassium salt alternatives. Use of phosphate and prebiotics in meat products, various types of fibers and starches in breads, and flavor enhancers and bitter blocking ingredients in cheese products were also among the current, common adoptable practices. Use of emerging technologies such as high pressure processing has also been an efficacious alternative to enhance the saltiness of various products while using lower amounts of sodium. 

With considerably lower cost of salt compared to nearly all other food ingredients, the cost associated with reducing sodium content of different products continues to be a major challenge for the private industry. Changes in organoleptic properties and microbiological food safety considerations will also continue to remain indispensable challenges for reduced- and low-sodium product development, requiring product specific innovation and optimization. Considering an array of health complications associated with excess dietary sodium, that many populations around the globe consume considerably more than the recommended amount of sodium, and that processed foods are the main source of dietary sodium intake in many nations, reducing the salt of manufactured food products will remain a major priority in short- and long-term public health interventions and an ethical duty for food entrepreneurs in the private industry. 
